# Routine use of laparoscopic techniques in daily practice improves outcomes after appendectomy

**DOI:** 10.1007/s00068-022-02125-4

**Published:** 2022-10-19

**Authors:** Tiia Mönttinen, Helmi Kangaspunta, Johanna Laukkarinen, Mika Ukkonen

**Affiliations:** 1 Department of Pediatric Surgery, New Children’s Hospital, Helsinki, Finland; 2 Faculty of Medicine and Health Technology, Tampere University, Tampere, Finland; 3 Department of Gastroenterology and Alimentary Tract Surgery, Tampere University Hospital, Teiskontie 35, 33521 Tampere, Finland

**Keywords:** Appendectomy, Appendicitis, Surgery, Laparoscopic appendectomy, Open appendectomy, Surgical resident, Senior surgeon, Experience

## Abstract

**Background:**

Appendectomy is the most common emergency operation and is often performed during on-call hours, when surgeons with different sub-specialties and levels of experience in emergency surgery operate on patients. However, little is known about the safety of the procedure when operations are performed by surgeons not regularly using standard laparoscopic techniques. Here we aim to assess variation in outcomes in patients operated on by surgeons with different levels of experience in laparoscopic surgery.

**Materials and methods:**

Consecutive patients undergoing appendectomy at Tampere University Hospital between September 1, 2014 and April 30, 2017 for acute appendicitis were included. The data were analyzed by level of experience among surgeons regularly performing laparoscopic surgery and by volume among surgeons performing over 30 appendectomies per year or fewer.

**Results:**

A total of 1560 patients underwent appendectomy, with 61% operated on by laparoscopic surgeons, and the rest by surgeons not habitually using laparoscopic techniques. Demographic characteristics, as well as share of patients with perforated appendicitis were similar in both groups. Morbidity was higher among those operated on by non-laparoscopic surgeons (6.1% and 3.0% *p* = 0.004), especially if appendicitis was complicated (18% and 5.6%, *p* < 0.001). Infectious complications were the most common. The risk of postoperative organ/space surgical site infections was higher among patients operated on by non-laparoscopic surgeons (3.5% vs. 1.4%, *p* = 0.006; Clavien–Dindo III–IV 2.0% vs. 0.7%, *p* = 0.030). Morbidity was 2.7% among those operated on by surgeons performing ≥ 30 appendectomies per year compared to 5.2% among those performing < 30 appendectomies per year. In multivariate analysis surgeon’s experience (*p* = 0.002; HR 2.32, 95% CI 1.38–3.90) and complicated disease (*p* < 0.001; HR 4.71; 95% CI 2.79–7.93) predicted higher morbidity.

**Discussion:**

According to our study, routine use of laparoscopic techniques in daily practice improves outcomes after appendectomy. In addition, a higher surgical volume correlates with improved outcomes.

## Introduction

Acute appendicitis is the most common indication for emergency gastrointestinal surgery [1–4] and laparoscopic appendectomy is the treatment of choice in acute appendicitis [5]. While appendectomy is among the basic procedures taught during surgical residency, specialists usually focus later on more complex operations and some perform only open surgeries. During on-call hours appendectomies are performed by surgeons with various sub-specialties and levels of experience in laparoscopic surgery.

Some earlier studies have compared the skills of surgical residents and experienced surgeons [6, 7], junior and senior residents [8], residents under supervision and those working independently [9] and also laparoscopic cholecystectomies performed by residents [9]. Those studies showed no significant differences in postoperative outcomes between residents and experienced surgeons, only one study found a difference in operating time [6].

While postoperative outcomes after open and laparoscopic appendectomy are well described in the literature, we found no studies comparing the results of surgeons with different levels of experience. Therefore we aimed here to study postoperative outcomes among patients operated on by surgeons performing open and laparoscopic surgery in their daily practice.

## Materials and methods

In this retrospective study consecutive patients operated on for acute appendicitis at Tampere University Hospital between September 1, 2014 and April 30, 2017 for acute appendicitis were included. Patients were identified from the institutional database by retrieving all surgeries associated with the Nordic Medico-Statistical Committee (NOMESCO) classification of surgical procedures (Version 1.13) code “JEA00” (appendectomy) and “JEA01” (laparoscopic appendectomy). Appendicitis was considered to be complicated if there was perforation and either peritonitis or abscess. Patients with negative appendectomies were excluded. Hospital records including surgical and histopathological reports were carefully retrieved individual for each patient.

Patients were operated on by in-house surgeons working in a high-volume tertiary care emergency hospital. The hospital catchment area covered health care in tertiary care services for approximately one million inhabitants. All emergency operations within the city of Tampere are performed in the study hospital. Attending surgeons were either residents (in gastrointestinal surgery, vascular surgery, urology, pediatric surgery or general surgery) with over two or more years’ experience in emergency surgery or consultant surgeons. In addition, a consultant gastrointestinal surgeon was available on-call at all times.

Patients were divided into two groups according to their surgeon’s experience in laparoscopic surgery. Laparoscopic surgeons (LS, 19 residents and 23 consultant surgeons) were those who performed laparoscopic surgery on a weekly basis in their daily practice and non-laparoscopic surgeons (NLS, 14 and 21) were those who did not use laparoscopy in their daily work. Gastrointestinal surgeons were either consultants or residents specializing into gastrointestinal surgery. The postoperative outcomes among all resident (33 surgeons) and consultant surgeons (44 surgeons) were also compared, likewise the outcomes among those surgeons performing lower and higher numbers of appendectomies each year.

The primary outcome measures were postoperative morbidity and mortality and the secondary outcome measures were operating time (minutes) and intraoperative bleeding (milliliters). Postoperative complications were defined and classified using the Clavien–Dindo (C–D) classification [10]. The survival data was obtained from the Finnish Population Register Centre. The results were analyzed separately in terms of whether the appendicitis was complicated or not. Follow-up data were available on all patients.

### Statistical analysis

All statistical analyses were performed using SPSS Statistics version 22 for Windows (IBM Corp, Armonk, NY, USA). *χ*^2^ or Fisher-tests were performed to compare categorical variables and Mann–Whitney *U*-test continuous variables. All tests used were two-tailed. Multivariate analysis (Binary Logistic Regression analysis, Enter method) was used to identify risk factors independently associated with worse outcomes. Statistical significance was set at a *p* value less than 0.05.

### Ethical aspects

The study was performed according to the Helsinki Declaration and institutional review board approval was obtained.

## Results

A total of 1560 patients (median age 35 [5–89] years; 48% female) underwent appendectomy during the study period. Sixty-one percent of the patients (*n* = 952) were operated on by LS, and the rest (39%, *n* = 608) by NLS. Demographic characteristics between these two groups were similar, as shown in Table 1. LS performed laparoscopic appendectomy more often than open appendectomy (70% vs. 89%, *p* < 0.001), while conversion rates from laparoscopic to open surgery were similar in both groups (1.4% vs. 2.0%, *p* = 0.351). The overall number of open appendectomies decreased during the study period being 18.4% at the beginning of the study period and 4.6% by the end.

**Table 1:** Patient- and operation-related characteristics

	Non-laparoscopic surgeons (*n* = 608)	Laparoscopic surgeons (*n* = 952)	*p* value
Age, median, years (min–max)	33 (5–83)	36 (6–89)	0.109
Sex, female	273 (45%)	474 (50%)	0.059
ASA I–II^a^	542 (89%)	858 (90%)	0.533
ASA III–IV^a^	66 (11%)	94 (10%)	0.533
Laparoscopic appendectomy	427 (70%)	847 (89%)	< 0.001
Open appendectomy	181 (29%)	105 (11%)	< 0.001
Conversion to open surgery	12 (2.0%)	13 (1.4%)	0.351
Uncomplicated appendicitis	464 (76%)	701 (74%)	0.235
Complicated appendicitis	144 (24%)	251 (26%)	0.235
Operation time, minutes (min–max)	41 (13–213)	37 (11–137)	< 0.001
Intraoperative bleeding, ml (min–max)	< 5 (0–300)	< 5 (0–510)	0.743
Morbidity	37 (6.1%)	29 (3.0%)	0.004
Clavien–Dindo I–II	24 (3.9%)	20 (2.1%)	0.031
Clavien–Dindo III–IV	13 (2.1%)	9 (0.9%)	0.051
SSSI^b^	4 (0.7%)	6 (0.6%)	0.947
O/SSSI^c^	21 (3.5%)	13 (1.4%)	0.006
O/SSSI (C–D^d^ III–IV)	12 (2.0%)	7 (0.7%)	0.030
Ileus	9 (1.5%)	1 (0.1%)	0.001
Bleeding	1 (0.2%)	2 (0.2%)	0.841
Pneumonia	2 (0.3%)	4 (0.4%)	0.777
Other	3 (0.5%)	3 (0.3%)	0.579
30-day mortality	0 (0.0%)	2 (0.2%)	0.258

^a^ASA: American Society of Anesthesiologists classification

^b^SSSI: Superficial surgical site infection

^c^O/SSI: Organ/space surgical site infection

^d^C–D: Clavien–Dindo classification

**Fig. 1:**
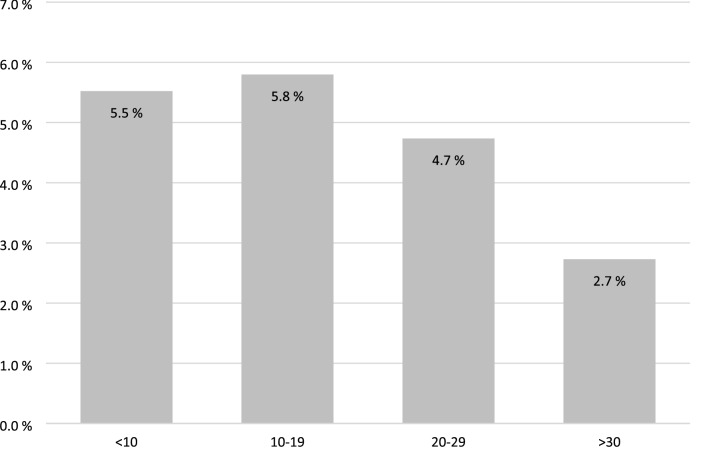
Comparison between surgeon volume and postoperative morbidity

Overall morbidity was higher among those operated on by NLS than those operated on by LS (6.1% vs. 3.0%, *p* = 0.004). Only two patients died within 30 days of surgery and neither of these deaths was due to appendicitis or surgery or morbidity-associated. Morbidity was higher in the NSL group after both laparoscopic (5.9% vs. 3.2%, *p* = 0.023) and open appendectomy (6.6% vs. 1.9%, *p* = 0.074). The most common postoperative complication was organ/space surgical site infection (2.2%). The risk of these infections after laparoscopic appendectomy was higher among those operated on by NLS than those operated on by LS (3.5% vs. 1.4%, *p* = 0.006; Clavien–Dindo III–IV 2.0% vs. 0.7%, *p* = 0.030). Postoperative outcomes after appendectomies are presented in Table 2 and Fig. 1.

**Table 2:** Outcomes after open and laparoscopic appendectomy

	Non-laparoscopic surgeons	Laparoscopic surgeons
Laparoscopic appendectomy	*n* = 427	*n* = 847
ASA I–II^a^	91%	91%
Complicated appendicitis	21%	25%
Operation time, minutes (min–max)	42 (13–144)	37 (11–137) (*p* < 0.001)
Intraoperative bleeding, ml (min–max)	< 5 (0–300)	< 5 (0–510)
Morbidity	5.9%	3.2% (*p* = 0.023)
SSSI^b^	0.7%	0.5%
O/SSSI^c^	4.2%	1.5% (*p* = 0.003)
O/SSSI (C–D^d^ III–IV)	2.6%	0.8% (*p* = 0.012)
Ileus	0.9%	0.1% (*p* = 0.027)
Bleeding	0.0%	0.2%
Pneumonia	0.0%	0.5%
Other	0.2%	0.4%
30-day mortality	0.0%	0.1%
Open appendectomy	*n* = 181	*n* = 105
ASA I–II^a^	85%	86%
Complicated appendicitis	31%	35%
Operation time, minutes (min–max)	34 (29–213)	30 (18–58)
Intraoperative bleeding, ml (min–max)	< 5 (0–100)	< 5 (0–10)
Morbidity	6.6%	1.9%
SSSI^b^	0.6%	1.9%
O/SSSI^c^	1.7%	0.0%
O/SSSI (C–D^d^ III–IV)	0.6%	0.0%
Ileus	2.8%	0.0%
Bleeding	0.6%	0.0%
Pneumonia	1.1%	0.0%
Other	1.1%	0.0%
30-day mortality	0.0%	1.0%

^a^ASA: American Society of Anesthesiologists classification

^b^SSSI: Superficial surgical site infection

^c^O/SSI: Organ/space surgical site infection

^d^–-D: Clavien–Dindo classification

Patients with uncomplicated and perforated appendicitis were further analyzed and the results are shown in Table 3. The outcome was worse if perforated appendicitis was operated on by NLS. In total 39 patients with complicated acute appendicitis (10%) suffered from postoperative complications. The respective shares in the LS and NLS groups were 5.6% and 18% (*p* < 0.001). Organ/space surgical site infections occurred more often if patients were operated on by NLS (12% vs. 3.6%, *p* = 0.003). The risk of postoperative ileus was also higher among those operated on by NLS (4.9% vs. 0.0%, *p* = 0.001).

**Table 3:** Outcomes after perforated and uncomplicated appendicitis

	Non-laparoscopic surgeons	Laparoscopic surgeons
Complicated appendicitis	(*n* = 144)	(*n* = 251)
ASA I–II^a^	82%	82%
Laparoscopic appendectomy	61%	85% (*p* < 0.001)
Operation time, minutes (min–max)	60 (31–213)	52 (17–137) (*p* = 0.001)
Intraoperative bleeding, ml (min–max)	< 5 (0–300)	< 5 (0–510)
Morbidity	18%	5.6% (*p* < 0.001)
SSSI^b^	1.4%	0.4%
O/SSSI^c^	12%	3.6% (*p* = 0.003)
O/SSSI (C–D^d^ III–IV)	5.6%	2.0%
Ileus	4.9%	0.0% (*p* = 0.001)
Bleeding	0.0%	0.4%
Pneumonia	0.7%	0.4%
Other	1.4%	0.4%
30-day mortality	2.1%	1.2%
Uncomplicated appendicitis	(*n* = 464)	(*n* = 701)
ASA I-II^a^	91%	93%
Laparoscopic appendectomy	73%	90%
Operation time, minutes (min–max)	40 (13–144)	32 (11–120) (*p* = 0.001)
Intraoperative bleeding, ml (min–max)	0 (0–200)	0 (0–200)
Morbidity	2.4%	2.4%
SSSI^b^	0.4%	0.7%
O/SSSI^c^	0.9%	0.6%
O/SSSI (C–D^d^ III–IV)	0.9%	0.3%
Ileus	0.4%	0.1%
Bleeding	0.2%	0.1%
Pneumonia	0.2%	0.4%
Other	0.2%	0.3%
30-day mortality	0.2%	0.4%

^a^ASA: American Society of Anesthesiologists classification

^b^SSSI: Superficial surgical site infection

^c^O/SSI: Organ/space surgical site infection

^d^C–D: Clavien–Dindo classification

**Fig. 2:**
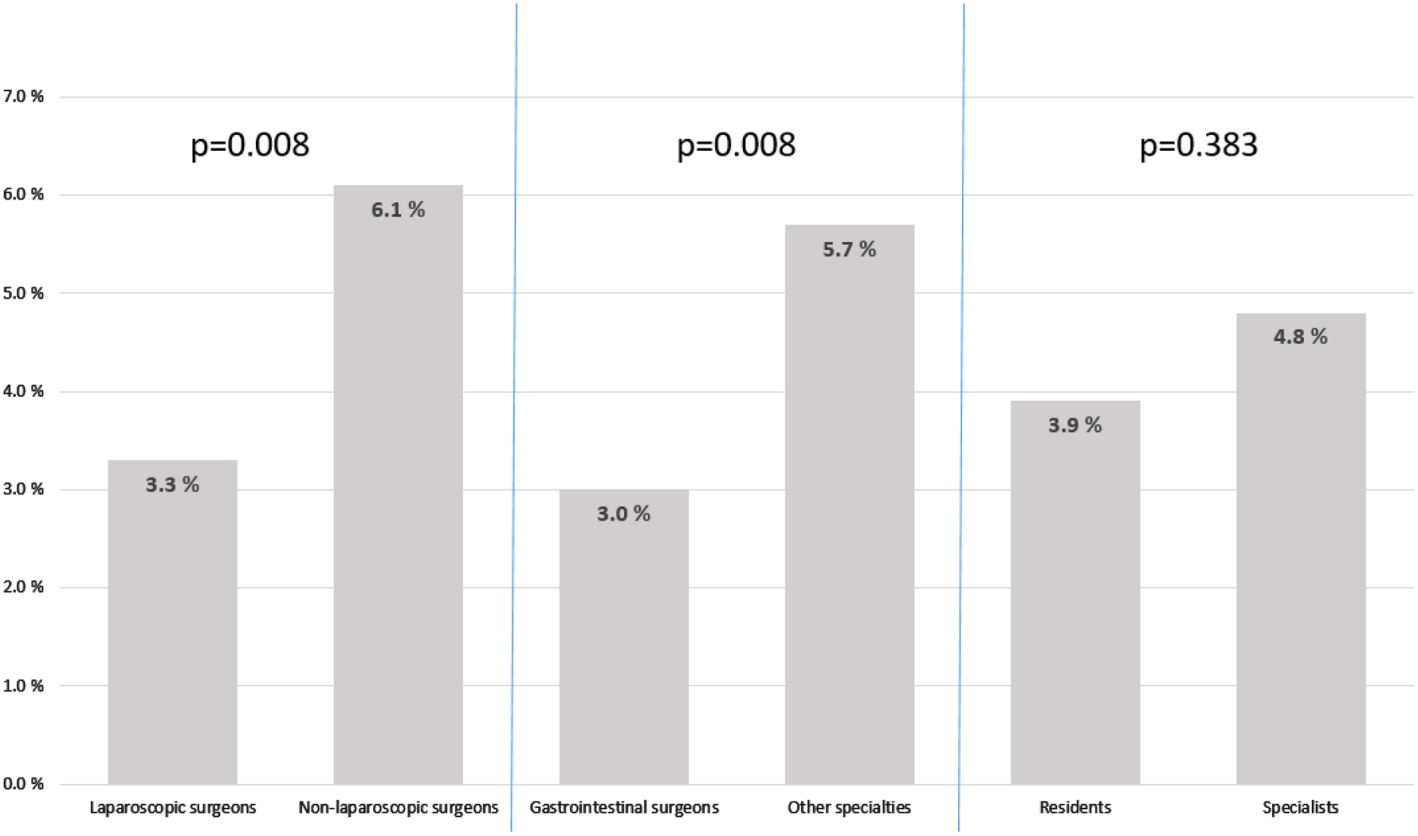
Postoperative morbidity after laparoscopic appendectomy in different patient groups

However, lower number of appendectomies performed per year was associated with higher morbidity. Morbidity was 2.7% among surgeons performing ≥ 30 appendectomies per year, compared to 5.2% among those performing fewer than 30 appendectomies per year, as shown in Fig. 1. Morbidity was similar when surgical residents and specialists were compared (3.9% vs. 4.8%, *p* = 0.383), as shown in Fig. 2. Gastrointestinal surgeons had the lowest rate of postoperative complications. In total, 2.8% (*n* = 22) of patients operated on by gastrointestinal surgeons suffered from postoperative complications.

When variables including ASA class (III or more), surgical technique, complicated appendicitis and surgeons’ experience were inserted into the multivariate analysis (binary logistic regression, enter method) surgeon’s experience (*p* = 0.002; HR 2.32, 95% CI 1.38–3.90) and perforated appendicitis (*p* < 0.001; HR 4.71; 95% CI 2.79–7.93) statistically significantly predicted higher morbidity, as shown in Table 4.

**Table 4:** Results of multivariate analysis

	HR	95% CI	*p* value
Perforated appendicitis	4.68	2.79–7.86	< 0.001
Non-laparoscopic surgeons	2.22	1.34–3.68	0.002
ASA score III or more^a^	1.25	0.56–2.79	0.588
Age 65 years or over	1.03	0.46–2.30	0.947

^a^ASA: American Society of Anesthesiologists classification

## Discussion

Appendectomy is the most common emergency operation and it is often performed during on-call hours when surgeons with different sub-specialties and levels of experience in emergency surgery operate on patients. However, little is known about the safety of appendectomy if the operation is performed by surgeons not routinely using standard laparoscopic techniques. According to our study, routine use of laparoscopic techniques in daily practice improves outcomes after appendectomy. The results were similar when residents and specialists were compared. In addition, a higher surgical volume correlated with improved outcomes.

Although significant human resources are needed to sustain 24/7 in-house or on-call surgeon availability, appropriate treatment of abdominal emergencies, including standard operations such as appendectomy, generates lower morbidity, higher patient satisfaction and cost-savings, and should be part of the services offered at least in tertiary-level facilities. While appendectomy in general is considered to be associated with a short-learning curve [11, 12], we have shown that maintaining skills requires continuous practice in laparoscopic techniques. Interestingly, those experienced in laparoscopic techniques also performed better when standard open technique was used. Even though appendectomy is considered to be among the most basic procedures every surgeon should be able to perform, whether this should indeed be so warrants discussion.

In cases of non-perforated acute appendicitis, the results were practically similar between both groups. The lack of specific and sensitive studies distinguishing between complicated and uncomplicated acute appendicitis creates some limitations [13]. One might propose that if a perforated appendicitis is diagnosed preoperatively, the procedure could be planned differently. Nevertheless, the problem arises during on-call hours, when those patients with complicated disease may not be able to wait as long as those with mild symptoms. Usually in mild cases patients can safely wait until the next morning. However, earlier surgery and symptom relief reduces length of hospital stay, improves patient satisfaction and also achieves cost-savings [14, 15].

We demonstrated that complicated procedures are associated with higher rate of postoperative infections. We were unable to ascertain whether non-laparoscopic surgeons used techniques causing a higher number of infectious complications and whether the rate of these could be reduced, for example, with simple education. A similar correlation with higher number of infections and low level of experience has been reported earlier in the literature. Whether we should or could focus on surgical education more is beyond the scope of this study. How emergency services should be provided remains also outside of the scope of this study.

There are some limitations in this study. We report retrospectively results from a single tertiary care center with all surgeons experienced in emergency surgery. However, we assume that in centers performing fewer emergency operations this difference in postoperative outcomes would be more marked. Among the strengths of this study was that follow-up data was available on all patients and also that all appendectomies were performed in the hospital concerned.

## Conclusion

According to our study, routine use of laparoscopic techniques in daily practice improves outcomes after appendectomy. Moreover, higher surgical volume correlates with improved outcomes.
